# Retrograde Migration of a Retained Radial Arterial Catheter Fragment: A Rare Perioperative Complication

**DOI:** 10.1155/cria/8704664

**Published:** 2025-12-07

**Authors:** Lotfi Rebai, Sarra Sammari, Olfa Faten, Firas Kalai, Sabrine Ben Brahem, Ichraf Ardhaoui

**Affiliations:** ^1^ Department of Anesthesiology and Critical Care, Trauma and Severe Burns Center, University of Tunis El Manar, Tunis, Tunisia, utm.rnu.tn

## Abstract

We report a rare case of inadvertent transection and retrograde intravascular migration of a radial arterial catheter fragment in a 52‐year‐old woman undergoing transcranial resection of a recurrent pituitary macroadenoma. The catheter had been inserted using the Seldinger technique and secured with three sutures. During catheter removal in the postoperative ward, an unexpected movement by the patient led to accidental transection and retention of the catheter’s distal portion within the radial artery. Initial bedside exploration was inconclusive. A computed tomography angiography (CTA) confirmed the presence of the retained catheter fragment approximately 3 cm proximal to the radial styloid. Surgical removal under general anesthesia was successful without complications. We review this rare complication in light of previously published cases and highlight preventative strategies.

## 1. Introduction

Radial artery cannulation is commonly performed in perioperative and critical care settings to allow continuous blood pressure monitoring and frequent arterial blood sampling. Despite being considered a relatively safe procedure, complications (albeit rare) can occur, including thrombosis, hematoma, infection, and, more uncommonly, ischemia or pseudoaneurysm formation [1].

One of the least‐reported complications is catheter fracture and intravascular migration, especially in a retrograde fashion. This rare event can result in foreign body embolization, vascular injury, or thrombotic complications if not promptly recognized and managed [2–5].

## 2. Case Presentation

A 52 year‐old woman, with a history of two prior transsphenoidal surgeries for pituitary macroadenoma, was scheduled for reoperation via a frontotemporal approach. General anesthesia and orotracheal intubation proceeded uneventfully. A 20‐G radial arterial catheter (Arterial LED Cath, VYGON, France) was placed in the left wrist using the Seldinger technique and secured with three nylon sutures (2/0): two lateral and one medial.

The surgical procedure was uneventful, and the patient was extubated 2 h later in the intensive care unit and subsequently transferred to the neurosurgical ward. During catheter removal, the attending nurse used a No. 11 scalpel to cut the sutures. A sudden involuntary movement of the patient’s hand led to accidental transection of the catheter and complete retention of the distal fragment within the radial artery.

**Figure 1:**
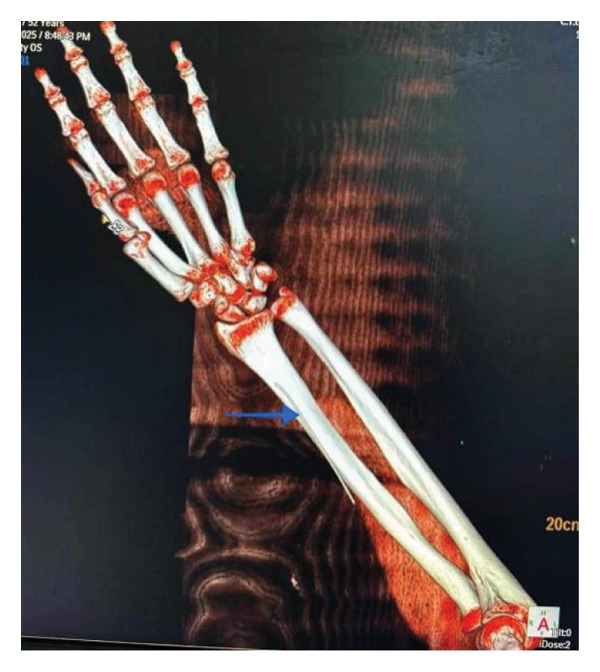
CTA of the left forearm showing retained catheter fragment (blue arrow) ∼3 cm proximal to radial styloid process.

On examination, the radial pulse was preserved, and there were no signs of distal ischemia. A bedside surgical exploration by a hand surgeon was inconclusive, prompting a computed tomography angiography (CTA) of the left upper limb. The scan revealed a catheter fragment located approximately 3 cm proximal to the radial styloid, suggesting retrograde migration (Figure 1). The patient was referred to vascular surgery, where the catheter was successfully removed under general anesthesia without postoperative complications.

## 3. Discussion

This case underscores a rare but significant complication of radial artery catheterization: complete transection followed by retrograde intravascular migration of a catheter fragment. While radial arterial lines are considered safe and are used extensively in perioperative monitoring and intensive care, they are not devoid of risk. Commonly described complications include thrombosis, infection, hemorrhage, or hematoma formation, but mechanical complications like catheter fracture and migration remain underreported, particularly in the adult population [1].

The mechanism of catheter transection in this case was iatrogenic and occurred during removal. The use of a scalpel in close proximity to the catheter shaft, especially in the presence of patient movement, carries an inherent risk of accidental laceration. This emphasizes a critical safety issue in clinical practice: although much attention is given to the insertion phase of arterial cannulation, catheter removal protocols are often overlooked in training and standard operating procedures.

Retrograde migration of an intravascular catheter fragment is extremely uncommon, as the natural arterial flow would typically favor distal embolization. However, in this case, the fragment was found approximately 3 cm proximal to the radial styloid, indicating a migration against arterial blood flow. Potential mechanisms for retrograde movement include the following:

• Vasospasm induced by manipulation or local trauma, temporarily altering flow dynamics.
• Negative pressure fluctuations caused by manual handling or repositioning of the limb.
• Local turbulence or reflux during catheter transection and attempted removal.

Previous case reports have described similar occurrences, supporting the notion that retrograde movement is physiologically plausible, albeit rare [6, 7].

CTA was instrumental in localizing the catheter segment with precision, enabling timely surgical planning. Although plain X‐rays may be sufficient for radiopaque fragments, CT or ultrasound guidance is preferred when fragments are small, embedded, or when vascular anatomy must be clearly delineated [8, 9]. The use of high‐resolution CTA in this case illustrates best practice for diagnosis and preoperative planning.

From a management standpoint, prompt extraction of retained intravascular foreign bodies is vital to prevent vascular thrombosis, distal ischemia, embolic events, and potential infection or endocarditis [4, 10]. Surgical retrieval remains the standard of care, particularly for arterial foreign bodies, though endovascular techniques have been explored in selected cases, primarily for venous systems or centrally located fragments [4].

In addition to the clinical lessons, this case highlights important system‐level concerns:

• The need for standardized catheter removal protocols, particularly in teaching hospitals.
• Adequate training of nursing staff and junior doctors on safe techniques for suture removal.
• The routine inspection of catheter integrity postextraction to detect incomplete removal.
• Preference for secure but easily removable fixation methods (e.g., adhesive dressings or transparent securement devices), particularly in high‐movement areas like the wrist.

Finally, this case contributes to the limited body of literature on arterial catheter fragment migration and should prompt further discussion and research into device design, safe removal techniques, and institutional protocols to mitigate such risks.

## Consent

This case report was submitted with the written consent of the patient.

## Conflicts of Interest

The authors declare no conflicts of interest.

## Funding

The authors received no financial support for this study.
